# Supplementary material for: Deep-ultraviolet ptychographic pocket-scope (DART): mesoscale lensless molecular imaging with label-free spectroscopic contrast
Source: eLight. 2026 Jan 7;6(1):1. doi: 10.1186/s43593-025-00103-y (PMC12774955; doi:10.1186/s43593-025-00103-y)
Supplement: Supplementary file 1 — Additional file 1. [file 43593_2025_103_MOESM1_ESM.pdf]

Supplementary Information for

Deep-ultraviolet ptychographic pocket-scope (DART): mesoscale lensless molecular imaging with label-free spectroscopic contrast

Ruihai Wang[1,&], Qianhao Zhao[1,&*], Julia Quinn[2], Liming Yang[1], Yuhui Zhu[1], Feifei Huang[1], Chengfei Guo[1], Tianbo Wang[1], Pengming Song[1], Michael Murphy[2], Thanh D. Nguyen[1], Andrew Maiden[3,4], Francisco E. Robles[5], and Guoan Zheng[1*]

[1]Department of Biomedical Engineering, University of Connecticut, Storrs, CT 06269, USA
[2]Immunopathology Laboratory, University of Connecticut Health Center, Farmington, CT 06030, USA
[3]Department of Electronic and Electrical Engineering, University of Sheffield, Sheffield, SYK S1 3JD, UK
[4]Diamond Light Source, Harwell, Oxfordshire OX11 0DE, UK
[5]Wallace H. Coulter Department of Biomedical Engineering, Georgia Institute of Technology and Emory University, Atlanta, GA 30332, USA
[&]These authors contributed equally
*Corresponding author: qianhao.zhao@uconn.edu or guoan.zheng@uconn.edu

Contents

Supplementary Figs. S1-S12……………………………………………………………………………… 2-13
Supplementary Note 1 and Figs. S13-16: Design and assembly of the DART system……………………………14-18
Supplementary Note 2 and Figs. S17-21: Ptychographic reconstruction with virtual states…………………… 19-25
Supplementary Videos S1-S3…………………………………………………………………………………… 26-28

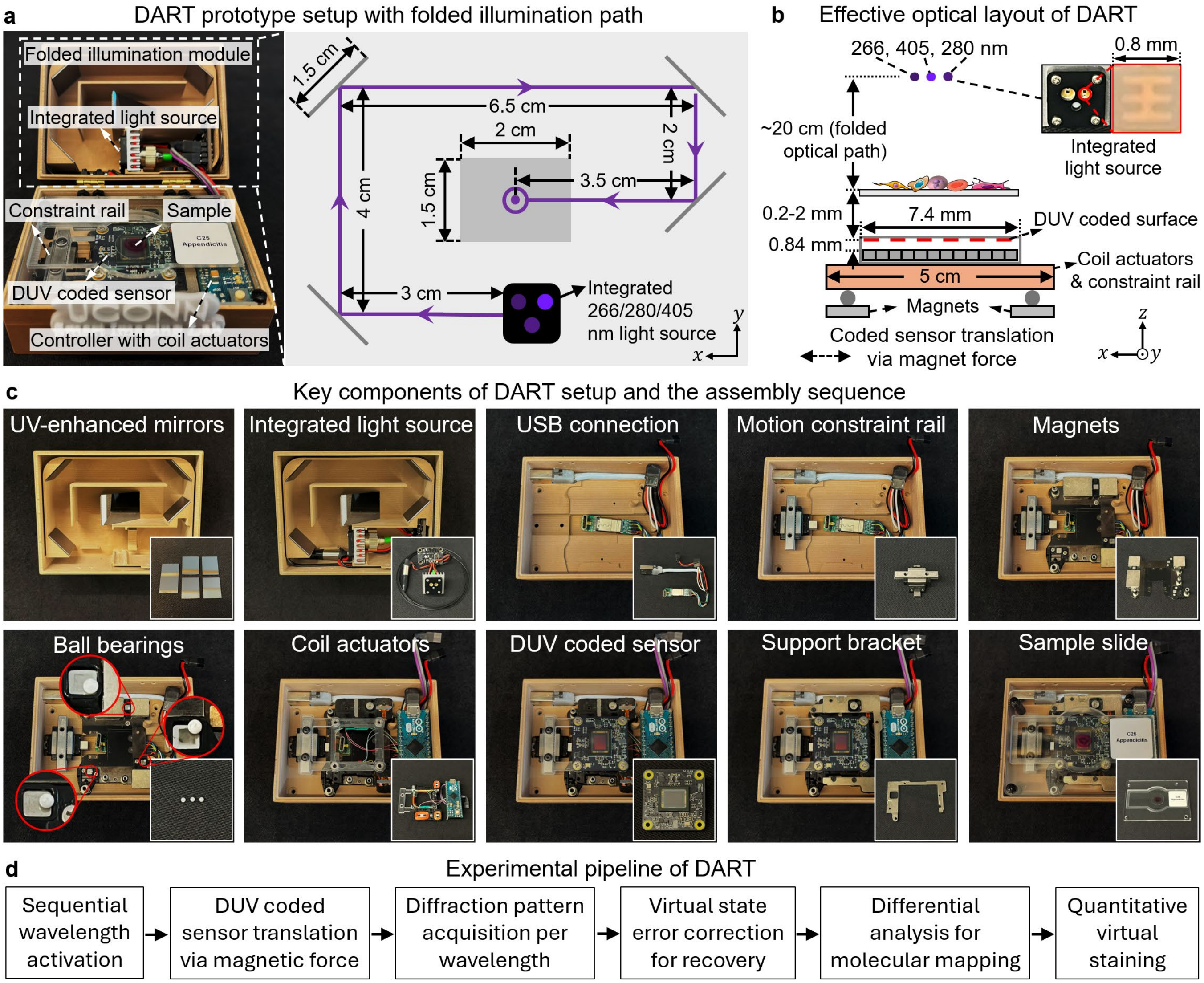

Supplementary Fig. S1 | Detailed experimental setup and pipeline of the DART system. (a) DART setup with a zoomed-in view of the folded illumination module. Five mirrors redirect the beam from the integrated light source along the folded optical path, extending the effective optical path to ~20 cm within a compact footprint. Labels mark the relative positions of the light source, sample, coded sensor, motion rail and control board. (b) Effective optical layout of DART showing the three sequentially-activated light sources, the sample-to-coded-surface distance, the fixed coded-surface-to-sensor separation, and the sensor translation mechanism driven by magnetic force from coil actuators. (c) Key components of the DART setup and assembly sequence. Top row shows individual optical and mechanical components: UV-enhanced mirrors with inset showing mirror array detail, integrated light source module with close-up of the three-wavelength configuration, USB connection board, motion constraint rail with precision bearings, and permanent magnets. Bottom row demonstrates the step-by-step assembly process: ball bearings installation for smooth motion, coil actuators integration for sensor control, DUV coded sensor mounting with inset showing the coded surface on the sensor, support bracket attachment, and final sample holder placement. (d) Experimental pipeline of DART showing the complete workflow from data acquisition to output.

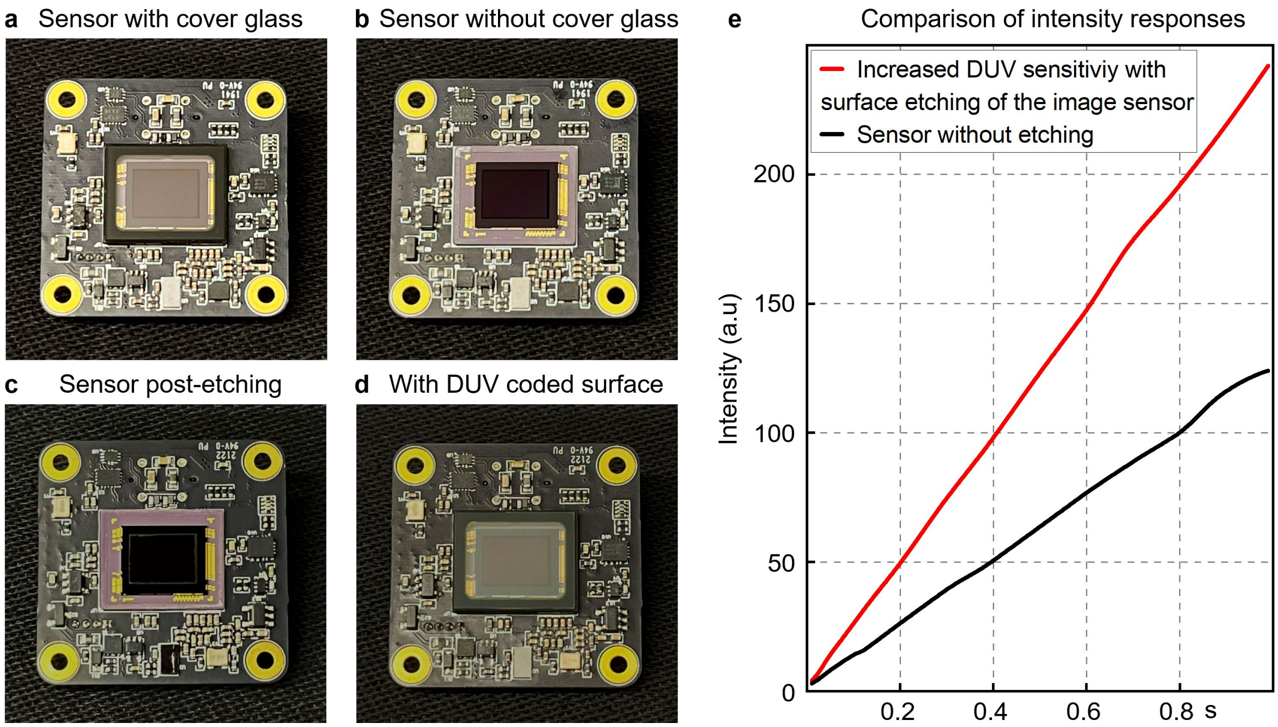

Supplementary Fig. S2 | Enhancement of DUV sensitivity through microlens etching on the image sensor. (a) The image sensor before any modifications, with a protective cover glass. (b) The image sensor after the removal of the cover glass, exposing the surface but without further treatment. (c) The image sensor after microlens etching to enhance DUV sensitivity by improving the light collection efficiency. (d) The sensor with the DUV-coded surface applied after microlens etching, optimized for DUV wavelength detection. (e) Comparing the DUV intensity responses of the sensor with microlens etching (red curve) and the sensor without etching (black curve). The sensor with etching shows twice increased DUV sensitivity.

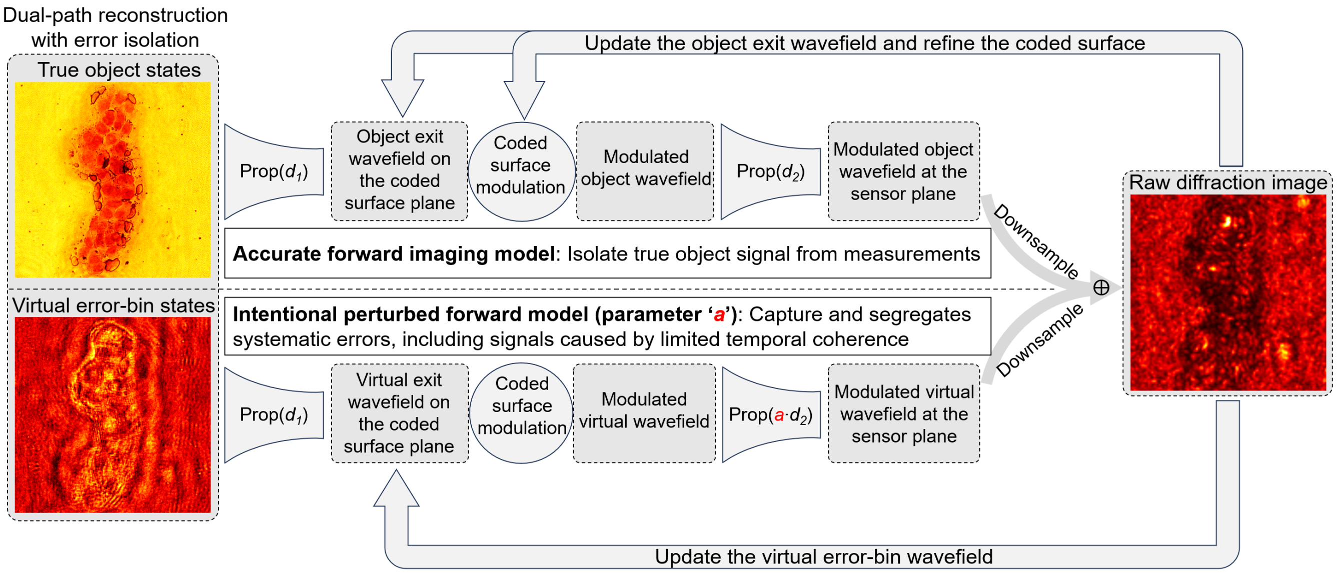

Supplementary Fig. S3 | Dual-path reconstruction with error isolation in DART. The top path represents the true object states and follows the accurate forward imaging model, where the object's exit wavefield is propagated to the coded surface plane, modulated, and then further propagated to the sensor plane to generate the modulated object wavefield. This model isolates the true object signal from the raw diffraction measurements. The bottom path represents the virtual error-bin states, which follow an intentionally perturbed forward model designed to capture and segregate systematic errors, including those caused by limited temporal coherence. The virtual exit wavefield undergoes a similar modulation and propagation process, but with an intentional wrong distance between the coded surface and the detector.

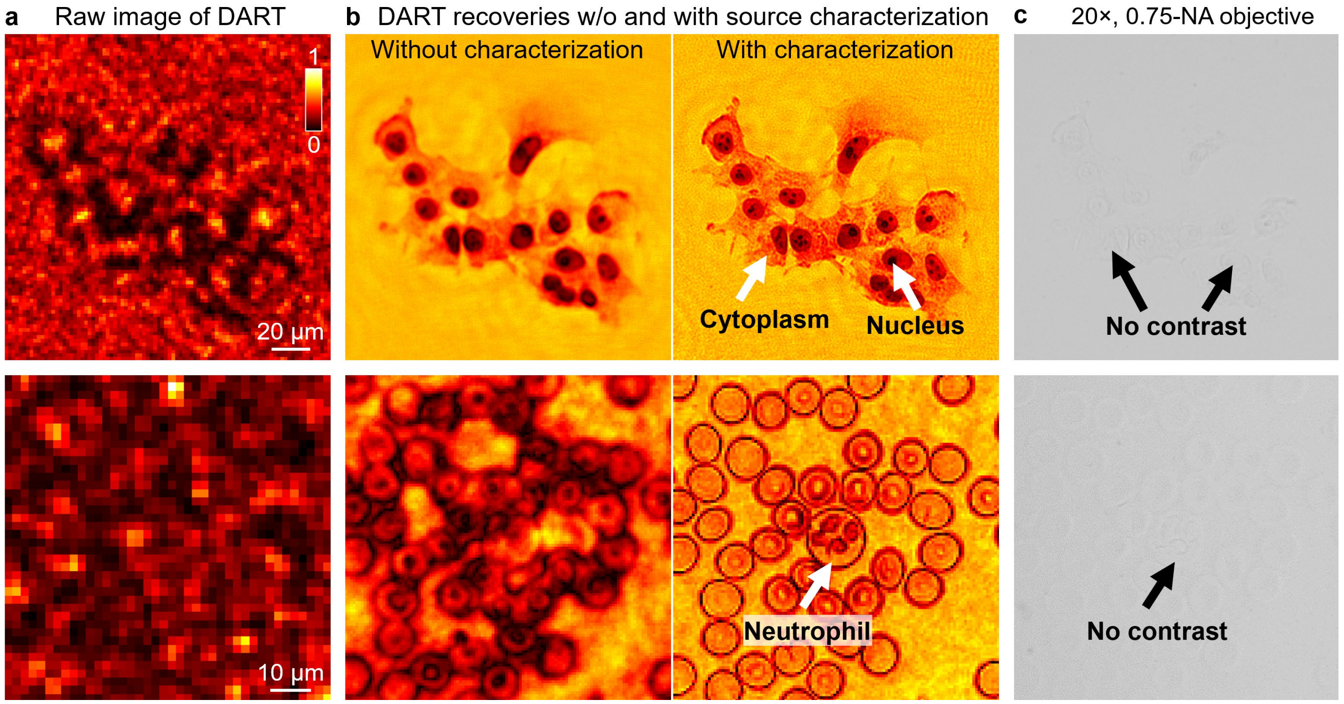

Supplementary Fig. S4 | Source characterization enhances DART imaging with partially coherent DUV light sources. (a) Raw DART diffraction images of biological samples before reconstruction. The upper panel shows unstained HEK-293 cell cultures and the lower panel shows unstained blood smear. (b) DART reconstructions without (left panel) and with (right panel) source characterization. With proper source characterization, we can enhance the resolution and reveal the sub-cellular structures. In the cell culture sample, the cytoplasm and nucleus are clearly resolved. In the blood smear sample, individual cells and a neutrophil are identified with high contrast. (c) Conventional 20×, 0.75-NA objective lens images of the same samples show no visible contrast, highlighting the advantage of DART with source characterization in recovering structural information without labels.

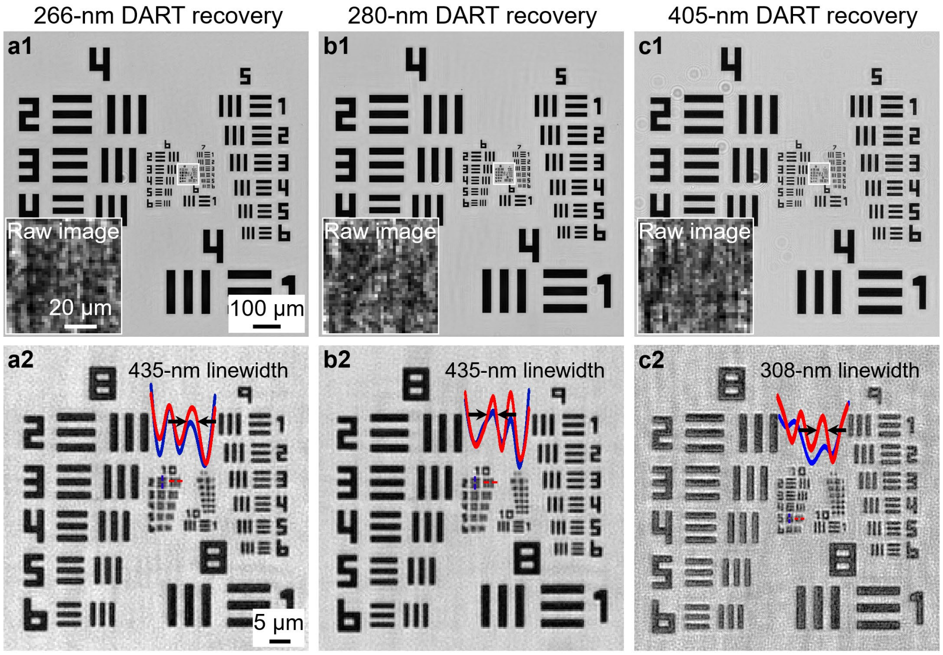

Supplementary Fig. S5 | Resolution characterization at 266 nm, 280 nm and 405 nm. (a1, b1, c1) DART reconstructions at 266 nm, 280 nm, and 405 nm wavelengths, respectively. Insets show the corresponding raw diffraction images at each wavelength. (a2, b2, c2) Close-up views of the recovered patterns from each wavelength, with intensity profiles across specific linewidths. The 266 nm and 280 nm reconstructions (a2, b2) resolve a 435-nm linewidth, while the 405 nm reconstruction (c2), utilizing a coherent 405 nm laser diode, results in slightly better resolution, resolving a 308-nm linewidth on the resolution target.

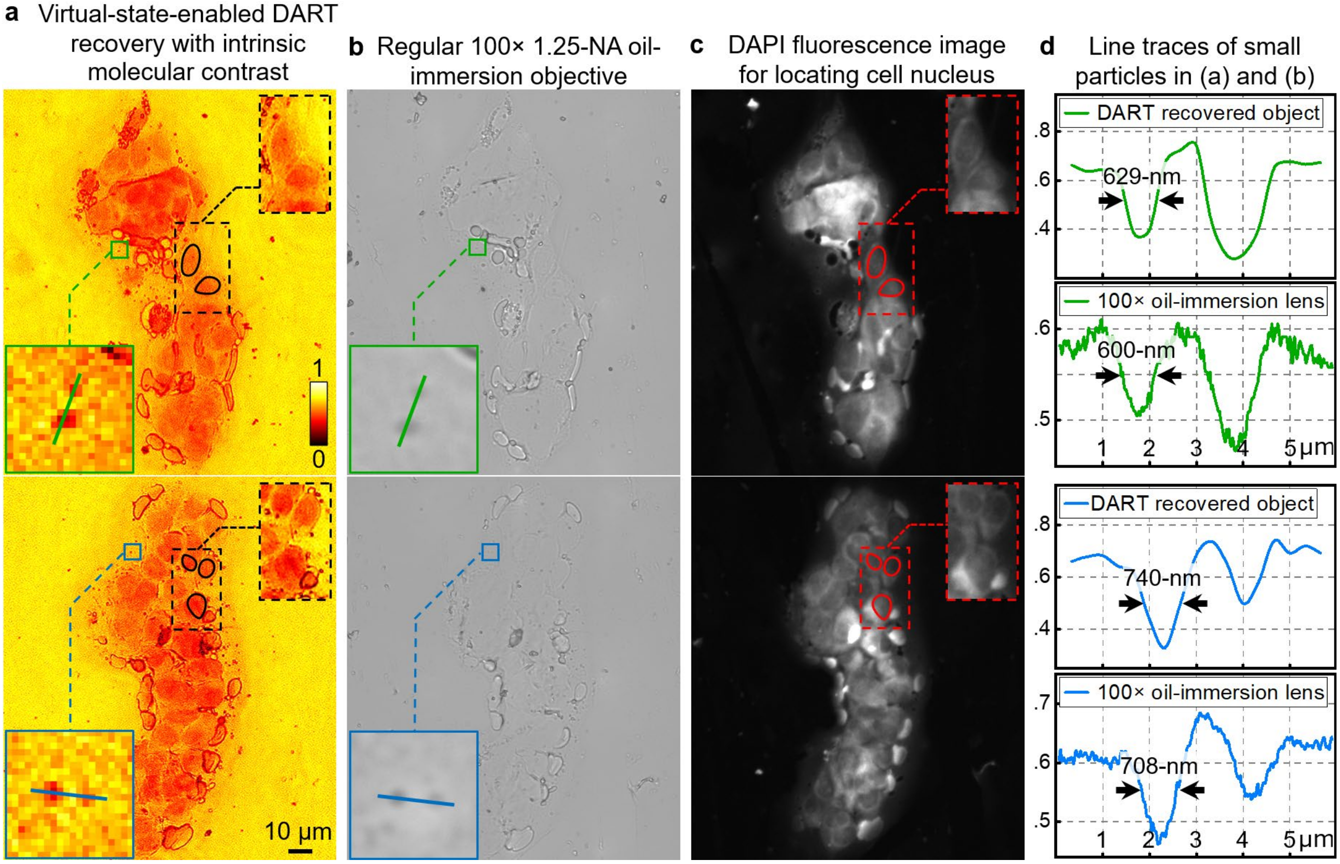

Supplementary Fig. S6 | Virtual-state-enabled DART imaging of an unstained lung FNA smear compared with conventional lens-based brightfield and fluorescence methods. (a) DART recovery with virtual-state correction showing intrinsic molecular contrast of the unstained lung FNA smear. Sub-cellular structures, including cytoplasm and nuclei, are clearly visible without the need for external labels or stains. The zoomed-in insets highlight the detailed resolution achieved by DART. (b) Regular 100×, 1.25-NA oil-immersion objective images of the same regions. (c) DAPI fluorescence imaging used to locate cell nuclei (red circles), confirming the structural features observed in the DART images. (d) Line traces comparing DART recovery (top and bottom panels) with the 100× oil-immersion objective (middle panels). The DART method resolves small particles as those with 100×, 1.25-NA oil-immersion objective lens.

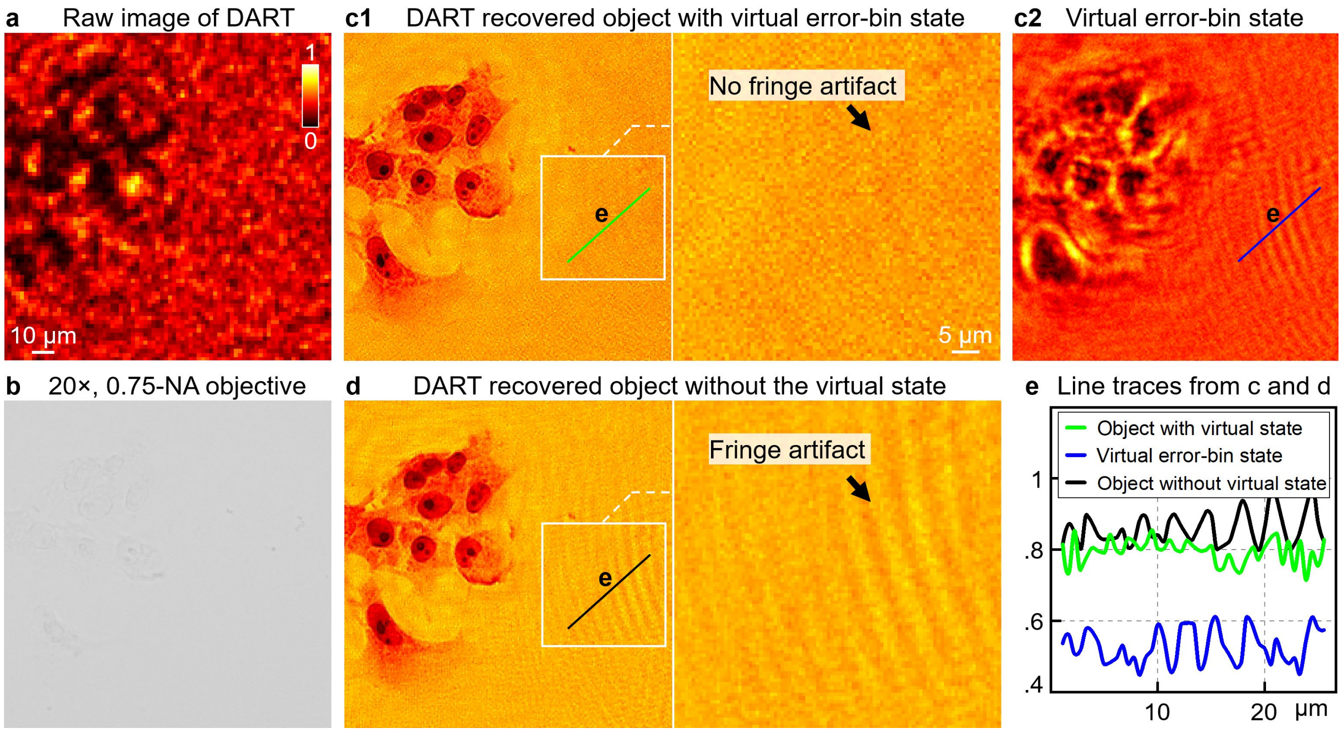

Supplementary Fig. S7 | Virtual state enables artifact isolation and removal in cell culture sample. (a) Raw DART diffraction image of unstained HEK 293 cell cultures. (b) Conventional 20×, 0.75-NA objective image of the same cells, showing no discernible contrast in the unstained sample. (c1) DART reconstructed object with the virtual error-bin state. The virtual error-bin state isolates and removes fringe artifacts caused by system imperfections. The zoomed-in inset (right) highlights the clean, artifact-free recovery. (c2) The virtual error-bin state itself, highlighting the regions of artifacts being isolated in the reconstruction process. (d) DART reconstructed object without the virtual error-bin state. The fringe artifacts are present in the reconstructed object. (e) A comparison of line profiles shows the improved fidelity of the object recovered with the virtual error-bin state (green) compared to the object without virtual state correction (black). The virtual error-bin state (blue) isolates artifacts, effectively improving the final object reconstruction quality.

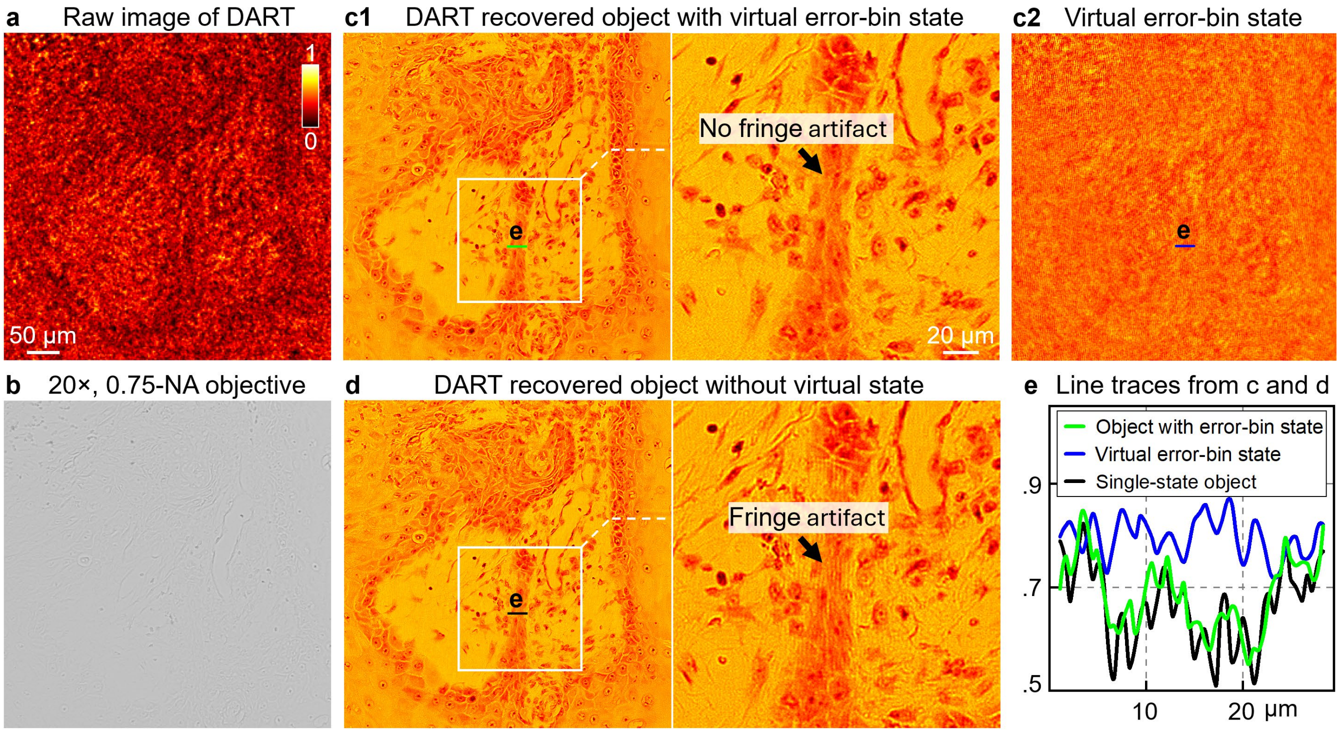

Supplementary Fig. S8 | Virtual state enables artifact isolation and removal in unstained pathology slide. (a) Raw DART diffraction image of unstained pathology slide. (b) Conventional 20×, 0.75-NA objective image of the slide, showing no discernible contrast in the unstained sample. (c1) DART reconstructed object with the virtual error-bin state. The virtual error-bin state isolates and removes fringe artifacts caused by system imperfections. The zoomed-in inset (right) highlights the clean, artifact-free recovery. (c2) The virtual error-bin state itself, highlighting the regions of artifacts being isolated in the reconstruction process. (d) DART reconstructed object without the virtual error-bin state. The fringe artifacts are present in the reconstructed object. (e) A comparison of line profiles shows the improved fidelity of the object recovered with the virtual error-bin state (green) compared to the object without virtual state correction (black). The virtual error-bin state (blue) isolates artifacts, effectively improving the final object reconstruction quality.

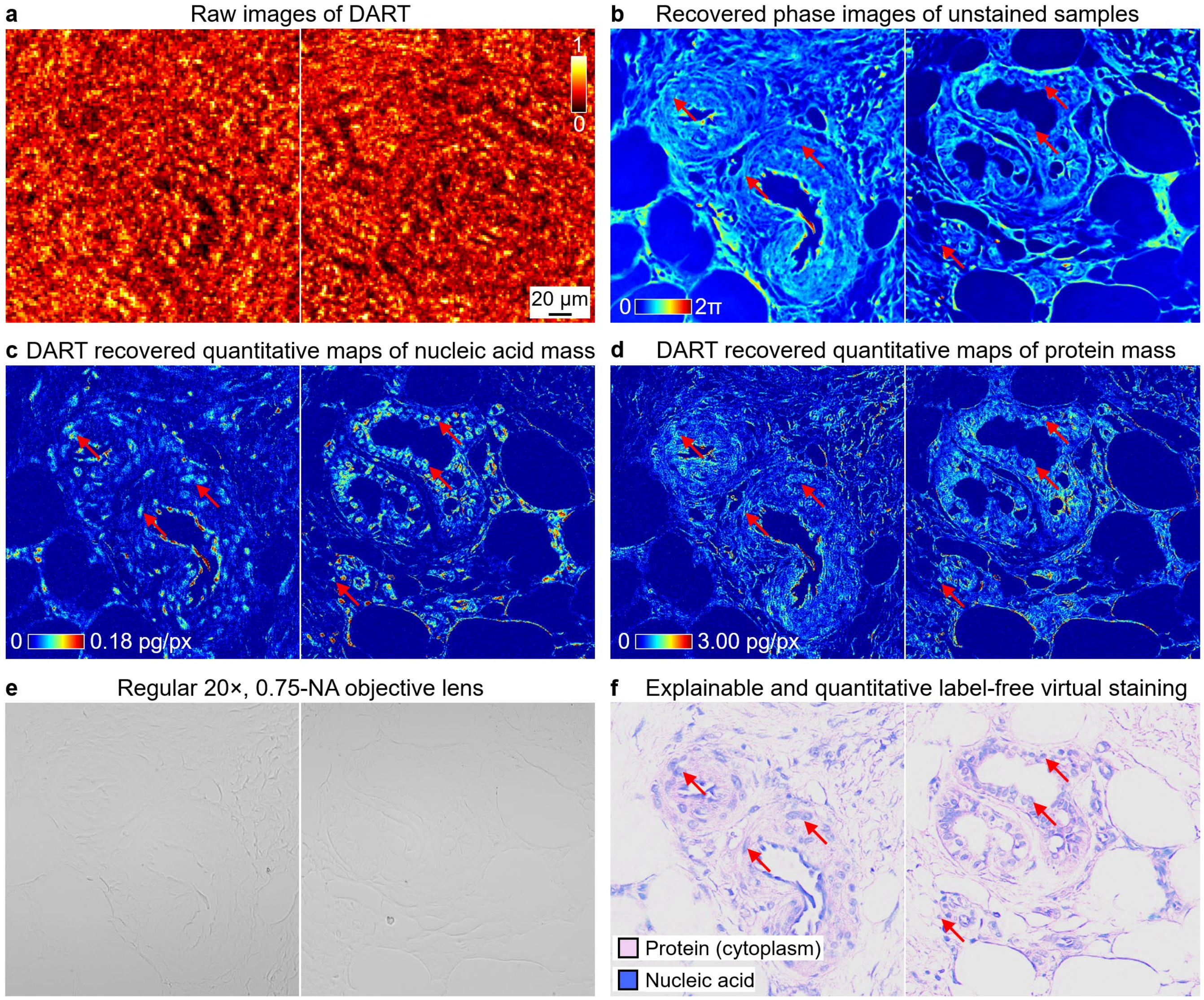

Supplementary Fig. S9 | DART enables clear identification of cellular structures in unstained samples compared to conventional brightfield and phase microscopy. (a) Raw DART diffraction measurements at 266-nm DUV wavelength. (b) Recovered phase images from DART, showing the unstained samples but offering limited contrast for resolving cellular structures, making it challenging to locate individual cells. (c, d) DART-recovered quantitative maps of nucleic acid mass (c) and protein mass (d), revealing the precise location and distribution of nucleic acids (e.g., nuclei) and proteins (e.g., cytoplasm) within the sample, enabling clear identification of cellular structures without the need for labeling. (e) Images captured using a regular 20×, 0.75-NA objective lens, showing minimal contrast and difficulty in identifying cells in the unstained sample. (f) Explainable and quantitative label-free virtual staining produced based on DART's recovered nucleic acid and protein mass maps, offering results comparable to traditional H&E stains. Nucleic acids (blue) and proteins (cytoplasm, pink) are clearly distinguished, providing reliable, quantitative cellular identification without external labels. Red arrows indicate key structures such as cell nuclei, which are clearly resolved with DART but not visible in the phase images.

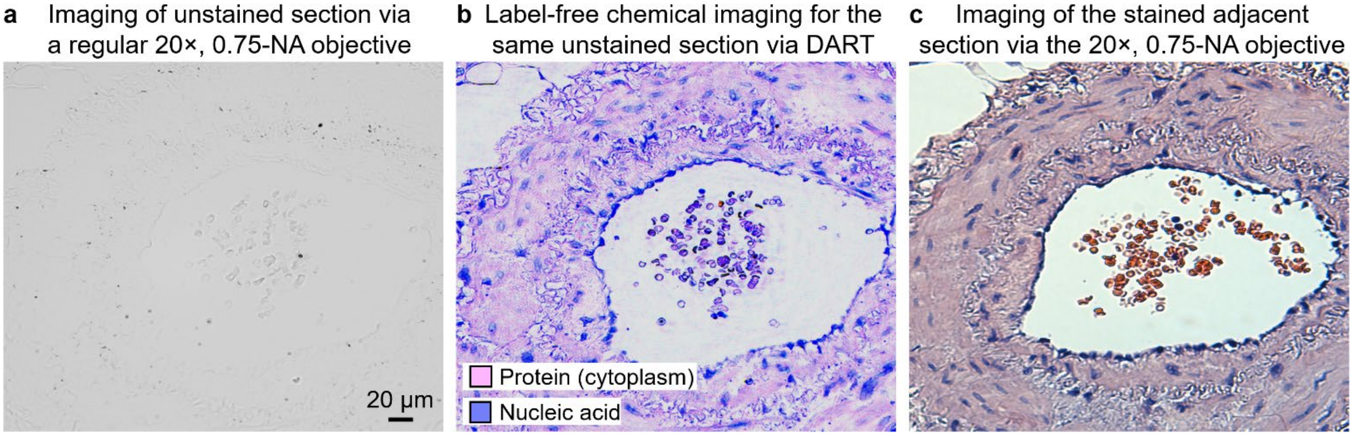

Supplementary Fig. S10 | Comparison of DART virtual staining with conventional brightfield microscopy of unstained and stained adjacent pathology sections. (a) Imaging of the unstained pathology section via a regular 20×, 0.75-NA objective, illustrating the low intrinsic contrast typical in unstained samples under conventional brightfield microscopy. (b) Label-free chemical imaging for the same unstained section via DART reconstruction. This result, presented as a virtual H&E stain, differentiates protein-rich cytoplasm (pink) and nucleic acid-rich nuclei (blue) based on their intrinsic DUV absorption signatures captured by DART. (c) Imaging of the stained adjacent section via the 20×, 0.75-NA objective after traditional H&E staining, providing a histological reference.

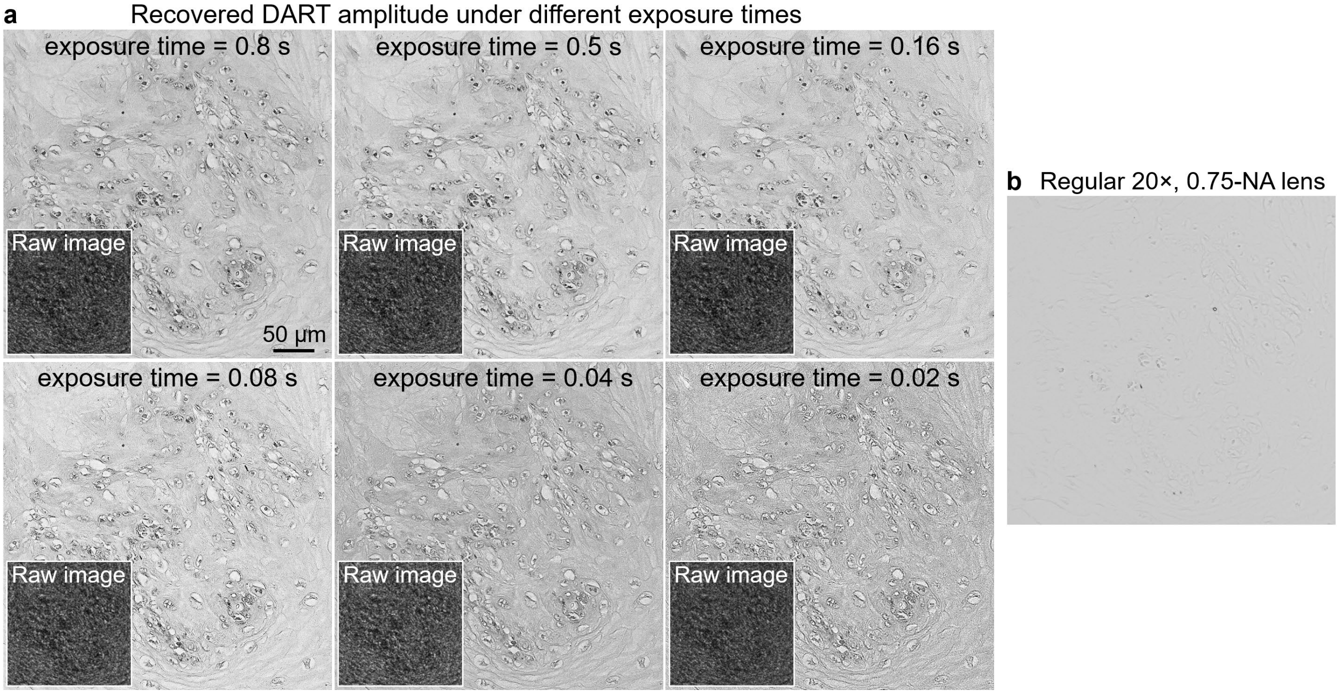

Supplementary Fig. S11 | Recovered DART amplitude under different exposure times. (a) DART amplitude images of an unstained pathology slide acquired at various exposure times, ranging from 0.8 seconds to 0.02 seconds. The corresponding raw diffraction images are shown as insets in each panel. Longer exposure times (e.g., 0.8 s) result in clearer reconstructions with improved signal strength, while shorter exposure times (e.g., 0.02 s) lead to noisier images. While meaningful structural information can still be recovered with shorter exposures, an exposure time of ~0.1 seconds is recommended for a typical DART implementation. (b) A conventional 20×, 0.75-NA objective lens image of the same region shows minimal contrast in the unstained sample.

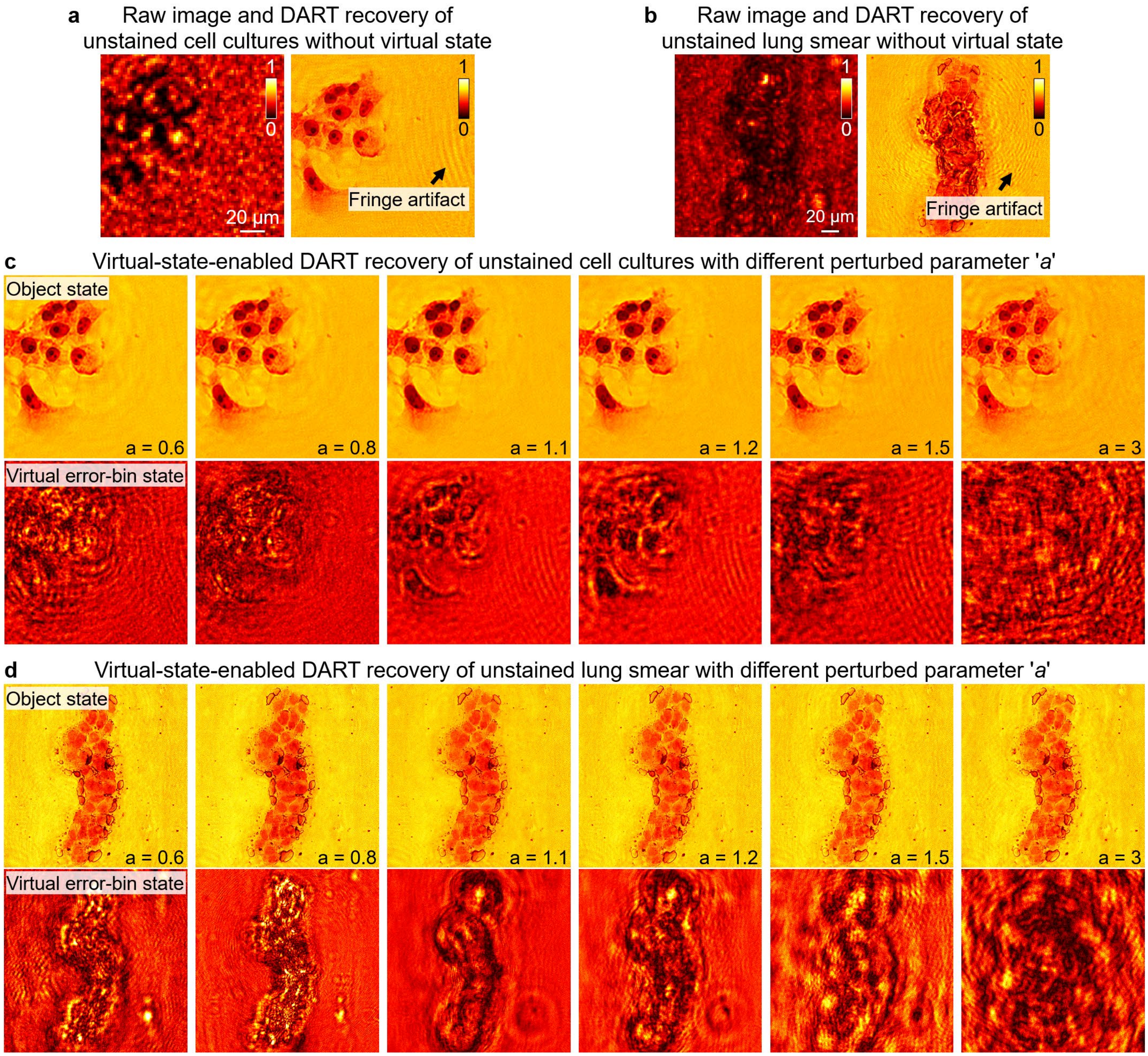

Supplementary Fig. S12 | Virtual-state-enabled DART recovery with intentional perturbed forward model for artifact isolation and removal. (a) Raw DART image and corresponding DART recovery of unstained cell cultures without virtual state correction. Visible fringe artifacts (black arrow), caused by system imperfections and partial coherence of the DUV light source, affect the image quality. (b) Raw DART image and corresponding DART recovery of an unstained lung smear without virtual state correction, showing fringe artifacts and low image fidelity. (c) Virtual-state-enabled DART recovery of the cell cultures with different values of the perturbed parameter '*a*' (0.6, 0.8, 1.1, 1.2, 1.5, and 3). The virtual error-bin state (bottom row) isolates noise and artifacts, improving the fidelity of the recovered object (top row). (d) Virtual-state-enabled DART recovery for the lung smear with different values of '*a*'. The object states (top row) show enhanced fidelity, while the virtual error-bin states (bottom row) isolate artifacts. In our DART implementation, we choose a =1.1 for effectively isolating errors from the diffraction measurements.

Supplementary Note 1: Design and assembly of the DART system

1. Assembly of the DART system

The DART system integrates high-resolution DUV imaging capabilities within a compact, handheld framework through meticulous coordination of optical, mechanical, and electronic components. This section details the systematic assembly process, emphasizing both structural elements and operational controls that enable label-free molecular imaging across diverse applications.

Optical path configuration. The assembly begins with establishing the optical path, optimized for deep-ultraviolet efficiency. UV-enhanced aluminum mirrors with ~97% reflectivity in the DUV region are strategically mounted (Supplementary Fig. S1c) to maximize light efficiency. Three light sources: a 266-nm DUV LED (Crystal IS KL265-50T-SM-WD), a 280-nm DUV LED (Nichia NCSU334A), and a 405-nm laser diode (D405-20, US-Lasers) are precisely positioned (Supplementary Fig. S1d) to enable multi-wavelength illumination for spectroscopic molecular contrast imaging.

Data and power integration. A custom USB interface is incorporated (Supplementary Fig. S1e) to facilitate data transfer and power delivery for all components, ensuring stable operation. This USB setup consolidates external connections, enhancing DART's compact and user-friendly design.

Mechanical motion control. Several engineered components are incorporated to enable precise motion control, necessary for high-resolution imaging. A motion constraint rail is installed (Supplementary Fig. S1f) to guide sample movement along defined paths while minimizing in-plane rotation. The design includes magnets (Supplementary Fig. S1g) and ball bearings (Supplementary Fig. S1h) within a voice coil actuator system inspired by sensor-shift technology used in smartphone cameras. This arrangement reduces friction, allowing smooth, controlled sensor movements with minimal mechanical resistance. Coil actuators (Supplementary Fig. S1i) enable fine control of sensor translation in both the X and Y directions.

Sensor and sample mounting. The DUV-coded sensor (Sony IMX 226) is modified to enhance DUV sensitivity by removing the protective cover glass and etching away the microlens array using photoresist stripper. A disorder-engineered coded surface, fabricated on thin fused silica substrate, is placed on top of the sensor to optimize ptychographic imaging (Supplementary Fig. S1j). A support bracket (Supplementary Fig. S1k) is added to provide structural stability, and a sample holder (Supplementary Fig. S1l) is secured to maintain biological specimen positioning during imaging. Hinges are installed to allow the smooth opening and closing of the device cover, preserving light-tight conditions when closed. This setup ensures that all components remain securely in place, even during handheld operation.

Final assembly. After completing the core structural and optical integrations, the DART prototype achieves a pocket-sized form factor suitable for various in-situ and field applications. The completed device (Supplementary Figs. S1a) demonstrates both portability and robustness, supporting sophisticated imaging functionalities in a handheld format, as demonstrated in Supplementary Videos S1 and S2.

2. Electronic control systems

The DART system employs the L293D motor driver, MCP4728 digital-to-analog converter (DAC), and Arduino Micro for precise control over motion and illumination. Supplementary Figs. S13-S15 detail these components and their role in DART's operation.

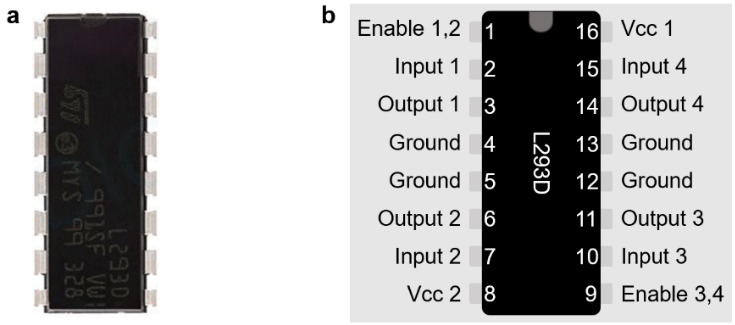

c L293D pin functions for controlling magnetic coil sets in a 2D translation stage

PIN		DESCRIPTION
NAME	NO.	
Enable 1,2	1	Activates channels 1 and 2 for controlling current through the magnetic coil set 1.
Input 1,2,3,4	2,7,10,15	Controls the direction of current through the coils, determining the polarity of the magnetic field.
Output 1,2,3,4	3,6,11,14	Outputs current to the coils based on input control, setting the magnetic field direction.
Enable 3,4	9	Activates channels 3 and 4 for controlling current through the magnetic coil set 2.
Ground	4,5,12,13	Provides a ground connection for the system.
Vcc 1	16	Supplies 5V for internal logic functions
Vcc 2	8	Provides power for driving the magnetic coils.

Supplementary Fig. S13 | L293D pin functions for controlling magnetic coil sets in a 2D translations stage. (a) The L293D breakout board. (b) Pinout diagram showing the 16-pin configuration. (c) Tabel of L293D pin functions, detailing control for the magnetic coil sets.

L293D motor driver. As illustrated in Supplementary Fig. S13, the L293D motor driver is a key component in controlling the precise movement of DART's 2D translation stage. This driver enables smooth, bidirectional motion along both the X and Y axes by regulating current flow through two sets of magnetic coils dedicated to each axis. Specifically, channels 1 and 2 of the L293D control the X-axis coils, while channels 3 and 4 handle the Y-axis coils, allowing DART to achieve controlled, multidirectional positioning essential for scanning samples effectively.

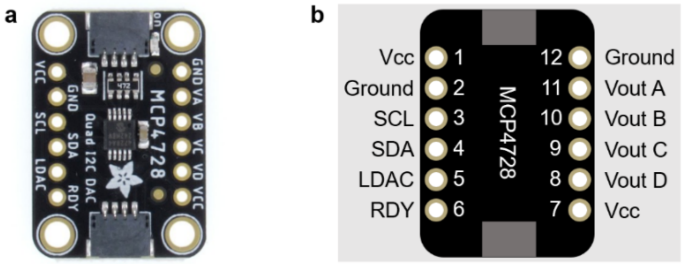

c MCP4728 pin functions for controlling LED states

PIN		DESCRIPTION
NAME	NO.	
Vcc	1,7	Power supply pins.
Ground	2,12	Provides a ground connection for the system.
SCL	3	Serial clock input for I2C communication. Synchronizes data transfer with the controller.
SDA	4	Serial data input/output for I2C communication. Transfer digital control signals for the light source.
LDAC	5	Output latching pin, used to latch the output when updating multiple DAC channels at once.
RDY	6	Ready pin. Pulled low when the DAC is busy writing to EEPROM, indicating it won't process new commands. Goes high when ready.
Vout A,B,C,D	8,9,10,11	Analog output pins for channels A, B, C, and D, used to control the on/off states of light source.

Supplementary Fig. S14 | MCP4728 pin functions for controlling LED states. (a) The MCP4728 breakout board. (b) Pinout diagram showing the 12-pin configuration (c) Tabel of MCP4728 pin functions, used to control light source states via I2C communication.

The L293D receives digital input signals from the Arduino Micro, which determines the direction of current flow through the coils. This setup enables the translation stage to move forward and backward along each axis. For fine-tuning the translation stage's speed, the Arduino supplies pulse-width modulation (PWM) signals to the Enable pins (pin 1 and 9), adjusting the strength of the current and thereby modulating the motor speed. This PWM-based speed control enhances the positioning accuracy of the stage, a critical factor when capturing diffraction patterns at multiple sample locations.

Each of the L293D's 16 pins is allocated for specific functions, including power input, ground, and signal control, all of which are vital to achieving optimal performance and stability. Supplementary Fig. S13c's pinout diagram clarifies the functional layout, demonstrating how the L293D's configuration allows for high-precision, flexible movement control across DART's imaging plane.

MCP4728 DAC for controlling the illumination light sources. Supplementary Fig. S14 details the MCP4728 digital-to-analog converter (DAC), a critical component in DART's illumination system that enables dynamic control over the DUV LEDs and 405 nm laser diode. With four analog output channels (VA, VB, VC, and VD), the MCP4728 DAC provides individualized voltage control for each light source. In this configuration, three channels are dedicated to adjusting the DUV LEDs, allowing fine modulation of light intensity to adapt illumination conditions to each sample's unique requirements.

The Arduino Micro communicates with the MCP4728 DAC through the I²C protocol, using the SDA and SCL pins to transmit digital commands that regulate the DAC's output voltages. This setup allows DART to seamlessly switch between various illumination modes and adjust light intensity in real-time.

The pinout diagram in Supplementary Fig. S14c offers a comprehensive view of the MCP4728's configuration, demonstrating how each pin contributes to precise control over DART's lighting system. This controlled illumination is crucial for enhancing image clarity and ensuring accurate, reliable data acquisition across different sample types.

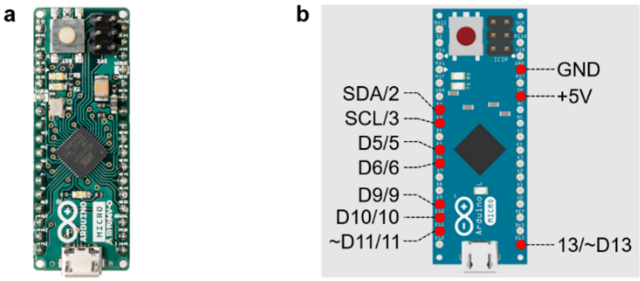

c

Arduino micro pin functions for controlling L293D and MCP4728

PIN		DESCRIPTION
NAME	NO.	
SDA	2	Serial data output for I2C communication. Transfer digital control signals to the MCP4728.
SCL	3	Serial clock output for I2C communication. Synchronizes data transfer with the MCP4728.
D5, D6	5,6	Digital outputs to L293D inputs 1 and 2, controlling direction for X-axis (coil set 1).
D9, D10	9,10	Digital outputs to L293D inputs 3 and 4, controlling direction for Y-axis (coil set 2).
~D11	11	PWM output to Enable 1,2 of L293D, controlling X-axis movement.
~D13	13	PWM output to Enable 3,4 of L293D, controlling Y-axis movement.
+5V	5V	Powers both L293D and MCP4728.
GND	GND	Common ground for L293D, MCP4728, and Arduino.

Supplementary Fig. S15 | Arduino Micro pin functions for controlling L293D and MCP4728. (a) The Arduino Micro controller. (b) Pinout diagram showing connections for I2C and motor control. (c) Tabel of Arduino Micro pin functions for controlling L293D and MCP4728.

Arduino Micro controller. The Arduino Micro serves as the central processing hub in DART, coordinating and synchronizing the operations of both the mechanical and illumination systems. Its role is crucial for maintaining the

precise control and responsiveness needed for high-resolution imaging, as well as for managing the complex interactions between the translation stage and the light sources. Supplementary Fig. S15 presents a detailed view of the pin connections and functions managed by the Arduino Micro within the DART system.

The Arduino Micro is configured to direct the movement of the 2D translation stage by controlling the L293D motor driver through dedicated digital pins, with pins D5 and D6 assigned to X-axis movement and pins D9 and D10 to Y-axis movement. By managing the direction of current flow in the motor driver, these pins enable the Arduino to precisely control forward and reverse motion along each axis, facilitating bidirectional movement of the translation stage. Additionally, for nuanced control over the stage's speed, the Arduino Micro delivers PWM signals to the Enable pins (pin 1 and 9) on the L293D. These PWM signals modulate the current strength applied to the motor driver, which adjusts the translation stage's speed accordingly. This level of precision is essential for capturing diffraction patterns at controlled positions across the sample, ensuring high-quality, consistent imaging.

In addition to controlling the translation stage, the Arduino Micro interfaces with the MCP4728 DAC to modulate DART's light sources, precisely regulating the intensity and timing of the DUV LEDs and 405-nm laser diode, key elements for DART's spectroscopic molecular imaging capabilities. Using the I²C protocol, the Arduino communicates with the DAC through pins D2 (SDA) and D3 (SCL), sending digital commands to adjust the DAC's output voltages. This ensures that the Arduino can precisely control the light intensity and on/off states of the light source. The pinout diagram in Supplementary Fig. S15c shows how each of the Arduino's pins is allocated, emphasizing its critical role in managing the entire DART system's operation.

Circuit layout. The circuit layout (Supplementary Fig. S16) illustrates the complex interconnections between DART's key components, showcasing the integration of power, I²C communication, and PWM control signals necessary for synchronized operation. The layout is designed to optimize data flow and power stability across the system, enabling efficient control of the DART's imaging and movement functionalities.

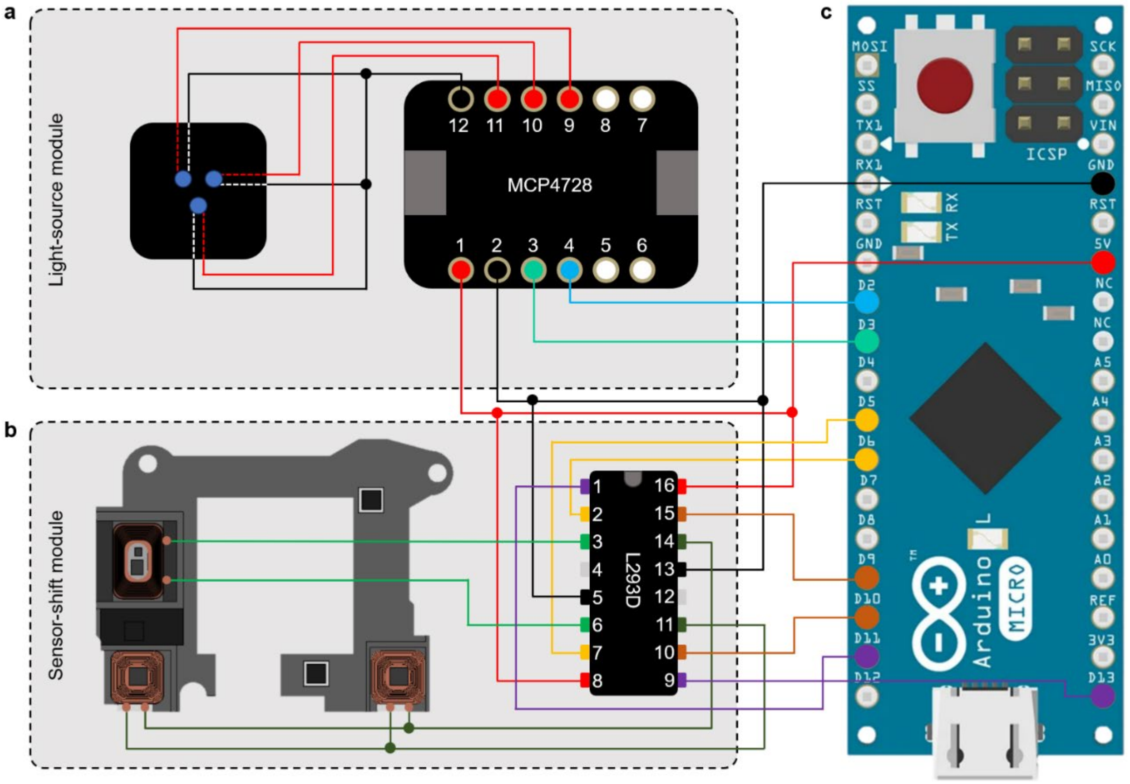

Supplementary Fig. S16 | Circuit Layout of the DART System. (a) Light-source module wiring, showing the connections between the light source and MCP4728 for controlling the light source states. (b) Sensor-shift module wiring, illustrating the connections between the magnetic coil set and L293D motor driver for controlling 2D movement. (c) Arduino Micro pin configuration, detailing the power, I2C, and PWM signal connections between the Arduino, MCP4728, and L293D.

In Supplementary Fig. S16a, the wiring for the light-source module connects the MCP4728 DAC to the DUV LEDs and the 405 nm laser diode. This configuration allows the Arduino Micro to precisely modulate light intensity and wavelength, adapting illumination conditions to the imaging requirements of each sample. By managing the voltage delivered to each light source through the DAC, the circuit enables fine-tuned illumination control, essential for achieving high-contrast images.

Supplementary Fig. S16b details the sensor-shift module wiring, which establishes the connections between the Arduino Micro and the L293D motor driver. This setup allows the Arduino to control the magnetic coils that drive the translation stage, enabling precise bidirectional movement in the X and Y axes. The PWM signals from the Arduino provide variable speed control, while digital outputs direct current flow, ensuring responsive, real-time adjustments in stage positioning. This circuit arrangement is critical for achieving stable, accurate sample scanning and capturing high-quality diffraction data.

Finally, Supplementary Fig. S16c provides a detailed map of the Arduino Micro's pin configuration, highlighting how it manages power, I^2C communication, and PWM signals to coordinate the entire system. This figure encapsulates the DART system's integration, demonstrating how each component's wiring contributes to the overall functionality of the device, ensuring smooth, synchronized operation for high-resolution imaging.

Supplementary Note 2: Ptychographic reconstruction with virtual states

The following MATLAB code simulates the generation of DART raw images and adds two different types of error to these raw images. High-fidelity object reconstruction is achieved through reconstruction with virtual state correction. The code consists of the following steps:

In step 1, we define the parameters for the imaging system, which are the same as our experimental setup.

```
%% Step 1: Set the parameters for the imaging system
phaseRangeObject = 1*pi;        % phase range of object
phaseRangeCS = 1*pi;            % phase range of coded surface
mag = 2;                        % up-sampling factor
centerPixel = round(mag/2);
originImSize = 256;
imSize = mag*originImSize;      % pixel number of groundtruth object
imSize0 = imSize/mag;           % pixel number of image sensor
waveLength = 0.266e-6;          % wavelength of deep-ultraviolet light source
pixelSize0 = 1.85e-6;           % image sensor pixel size
pixelSize = (1.85e-6)/mag;      % ground truth object pixel size
d1 = 300e-6;                    % the distance between the coded surface and object
d2 = 840e-6;                    % the distance between the coded surface and image
                                sensor
sourceDistance = 2000e-6;       % the distance between the light source and object
arraySize = 10;
imNum = arraySize^2;            % total number of raw images
stepSize = 1*mag;               % scanning step size between each 2 measurements
```

In step 2, we generate the complex input object, coded surface profile and error as shown in Supplementary Fig. S17. The image's size is 512 by 512 pixels. The amplitude of the input object is normalized, and the input phase is set from -pi/2 to pi/2. This complex high-resolution object is stored in 'objectGT' and the coded surface profile is stored in 'cs'. In our implementation, we define two types of error. One type of error is spatially invariant static error, represented by the built-in MATLAB image 'pears'. The other type is spatially variant shifted error, shifting with the sample, and is represented by the built-in 'mandrill' image. After normalizing both images, the error intensity is adjusted by modifying the coefficients that control the strength of the error image added to the raw images. They are stored in the variables 'errorInvariant' and 'errorVariant', respectively.

```
%% Step 2: Generate the complex input object and define errors
objectAmplitude = single(imread('cameraman.tif'));
objectAmplitude = imresize(objectAmplitude,[originImSize,originImSize]);
objectAmplitude = objectAmplitude/max(objectAmplitude(:));
objectAmplitude = imresize(objectAmplitude,mag);
objectPhase = single(imread('westconcordorthophoto.png'));
objectPhase = imresize(objectPhase,[originImSize,originImSize]);
objectPhase =phaseRangeObject*(objectPhase/max(objectPhase(:)))-phaseRangeObject/2;
objectPhase = imresize(objectPhase,mag);
objectGT = objectAmplitude.*exp(1i.*objectPhase);
csAmplitude = rand(imSize,imSize) + 0.3;
csPhase = rand(imSize,imSize);
csPhase = phaseRangeCS*csPhase-phaseRangeCS/2;
cs = csAmplitude.*exp(1i.*csPhase);
```

```
pears = imread("pears.png");
error = double(imresize(rgb2gray(pears),size(objectGT)));
errorInvariant = error./max(error (:)).*2;
clear error
load ('mandrill','X')
error = double(imresize(X,size(objectGT)));
errorVariant = error./max(error (:)).*2;
figure(1);
subplot(2, 3, 1); imshow(abs(objectGT), []); title({'Object amplitude', '(Ground truth)'})
subplot(2, 3, 2); imshow(abs(cs), []); title({'Coded surface amplitude', '(Ground truth)'});
subplot(2, 3, 3); imshow(errorInvariant, []); title({'spatially invariant error', '(Ground truth)'});
subplot(2, 3, 4); imshow(angle(objectGT), []); title({'Object phase', '(Ground truth)'})
subplot(2, 3, 5); imshow(angle(cs), []); title({'Coded surface phase', '(Ground truth)'});
subplot(2, 3, 6); imshow(errorVariant, []); title({'spatially variant error', '(Ground truth)'});
```

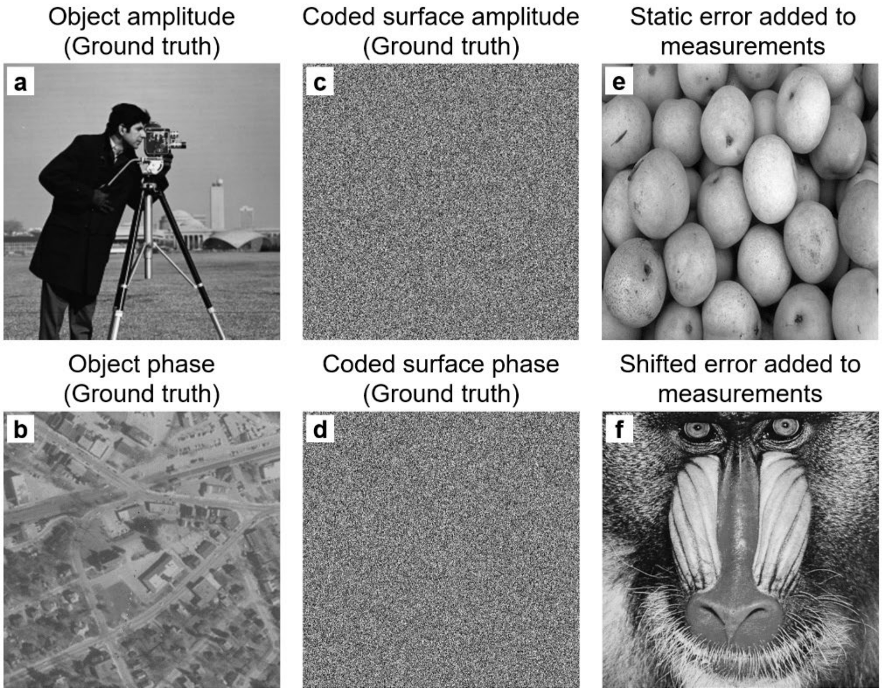

Supplementary Fig. S17 | Overview of the elements generated in Step 2. (a) Amplitude of the high-resolution object. (b) Phase of the high-resolution object. (c) Amplitude of the coded surface profile. (d) Phase of the coded surface profile. (e) Spatially invariant static error. (f) Spatially variant shifted error.

In step 3, we initialize subpixel shift parameters and propagation parameters. In this step, we define the frequency domain coordinates. It is important to note that when defining the propagation function, a perturbed parameter 'a' is introduced for the virtual state, along with the corresponding propagation function for the virtual state.

```
%% Step 3: Generate the frequency domain coordinates and propagation phase factor.
k0 = 2*pi/waveLength;
kmax = pi/pixelSize;
kxm = gpuArray.linspace(-kmax,kmax,imSize);
kym = gpuArray.linspace(-kmax,kmax,imSize);
[kxmGrid,kymGrid] = meshgrid(kxm,kym);
kzmGrid = single(sqrt(complex(k0^2- kxmGrid.^2- kymGrid.^2)));
```

```
50.fy0 = ifftshift(gpuArray.linspace(-floor(imSize0/2),ceil(imSize0/2)-1,imSize0));
51.fx0 = ifftshift(gpuArray.linspace(-floor(imSize0/2),ceil(imSize0/2)-1,imSize0));
52.[ fx0Grid,fy0Grid] = meshgrid(fx0,fy0);
53.fy = ifftshift(gpuArray.linspace(-floor(imSize/2),ceil(imSize/2)-1,imSize));
54.fx = ifftshift(gpuArray.linspace(-floor(imSize/2),ceil(imSize/2)-1,imSize));
55.[fxGrid,fyGrid] = meshgrid(fx,fy);
56.propD1 = exp(1i.*d1.*real(kzmGrid)).*exp(-abs(d1).*abs(imag(kzmGrid))).*((k0^2-kxmGrid.^2-kymGrid.^2)>=0);
57. propD1Reverse = exp(1i.*(-d1).*real(kzmGrid)).*exp(-abs((-d1)).*abs(imag(kzmGrid))).*((k0^2-kxmGrid.^2-kymGrid.^2)>=0);
58. propD2 = exp(1i.*d2.*real(kzmGrid)).*exp(-abs(d2).*abs(imag(kzmGrid))).*((k0^2-kxmGrid.^2-kymGrid.^2)>=0);
59. propD2Reverse = exp(1i.*(-d2).*real(kzmGrid)).*exp(-abs((-d2)).*abs(imag(kzmGrid))).*((k0^2-kxmGrid.^2-kymGrid.^2)>=0);
60.a = 1.2;
61.propD3 = exp(1i.*(d2.*a).*real(kzmGrid)).*exp(-abs(d2.*a).*abs(imag(kzmGrid))).*((k0^2-kxmGrid.^2-kymGrid.^2)>=0);
62. propD3Reverse = exp(1i.*(-d2.*a).*real(kzmGrid)).*exp(-abs((-d2.*a)).*abs(imag(kzmGrid))).*((k0^2-kxmGrid.^2-kymGrid.^2)>=0);
```

In step 4, we use nine tilted plane waves from different angles to simulate the partially coherent light source.

```
%% Step 4: Generate illumination pattern.
63.xIllum = (-imSize/2+1)*pixelSize:pixelSize:imSize/2*pixelSize;
64.yIllum = (-imSize/2+1)*pixelSize:pixelSize:imSize/2*pixelSize;
65.[xIllumGrid,yIllumGrid] = meshgrid(xIllum,yIllum);
66.source = zeros(imSize,imSize);
67.central = [imSize/2,imSize/2];
68.radius = 2;
69.for x = -radius:1:radius
70.for y = -radius:1:radius
71.if abs(x) == 1 || abs(y) == 1
72.else
73.source(central(1)+x,central(2)+y) = 1;
74.end
75.end
76.end
77.centerX = 0;
78.centerY = 0;
79.[sx,sy] = find(source == 1) ;
80.sx = sx - imSize/2 +centerX;
81.sy = sy - imSize/2 +centerY;
82.numberIllum = length(sx);
83.illum = zeros(imSize,imSize, numberIllum);
84.for illumIndex = 1: numberIllum
85.xIllumNA = (sx(illumIndex)*pixelSize0)/sqrt((sourceDistance)^2 + (sx(illumIndex)*pixelSize0)^2 + (sy(illumIndex)*pixelSize0)^2 );
86. yIllumNA = (sy(illumIndex)*pixelSize0)/sqrt((sourceDistance)^2 + (sx(illumIndex)*pixelSize0)^2 + (sy(illumIndex)*pixelSize0)^2 );
```

```
kxIllum = - xIllumNA.*k0;
kyIllum = - yIllumNA.*k0;
illum(: , : , illumIndex) = exp(1j.* kxIllum.* xIllumGrid +1j.* kyIllum.* yIllumGrid);
end
```

In step 5, we generate a spiral sequence for object scanning. The object shifts along the 18-by-18-pixel spiral path, as shown in Supplementary Fig. S18. The x- and y- positions are stored in 'locX' and 'locY'.

```
%% Step 5: Generate the spiral sequence.
routeSpiral = spiral(arraySize);
locX = zeros(1,imNum);
locY = zeros(1,imNum);
for i = 1:imNum
[locXTemp,locYTemp] = find(routeSpiral==i);
locX (1,i) = stepSize*(locXTemp-round(arraySize/2));
locY (1,i) = stepSize*(locYTemp-round(arraySize/2));
end
figure(2);plot(locX,locY,'*-');title('scanning route');
```

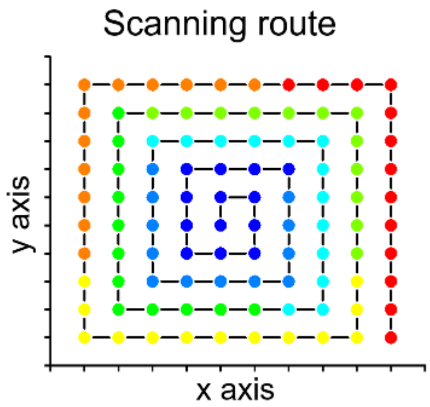

Supplementary Fig. S18 | Scanning route for the imaging system.

In step 6, we generate the low-resolution image sequence by scanning the high-resolution object to different positions. This scanning is achieved by shifting the object, which we accomplish by multiplying an equivalent phase factor 'Shift' to the spectrum of the object. The shifted object then interacts with tilted plane waves from various angles. After this interaction, the wavefront undergoes modulation by a coded surface. The wavefront intensities are incoherently mixed on the sensor plane. The sensor then captures this intensity, and two sets of error are added to the intensity, resulting in the final raw images, as shown in Supplementary Fig. S19.

```
%% Step 6: Generate low-resolution measurements.
imRaw = gpuArray.zeros(imSize0,imSize0,imNum,'single');
for i = 1:imNum
shiftRaw = exp(-1j*2*pi.*( fxGrid.*-locX (i)/imSize0+ fyGrid.*-locY (i)/imSize0));
objectShift = ifft2(fft2(objectGT).* shiftRaw);
objectShiftIllum = objectShift.*illum;
objectProp = ifft2(ifftshift(propD1.*fftshift(fft2(objectShiftIllum)))) ;
waveSensorPlane = ifft2(ifftshift(propD2.*fftshift(fft2(objectProp.*cs))));
intenSensorPlane = conv2(sum(abs(waveSensorPlane).^2,3),ones(mag,mag),'same');
errorVariantShift = ifft2(fft2(errorVariant).*shift);
```

```
109.imRaw(:,:,i) = intenSensorPlane(centerPixel:mag:end,centerPixel:mag:end) +…
(errorInvariant (centerPixel:mag:end,centerPixel:mag:end))+…
(errorVariantShift (centerPixel:mag:end,centerPixel:mag:end));
110.end
111.figure(3);imshow(abs(imRaw(:,:,1)),[]);title('1st captured image')
```

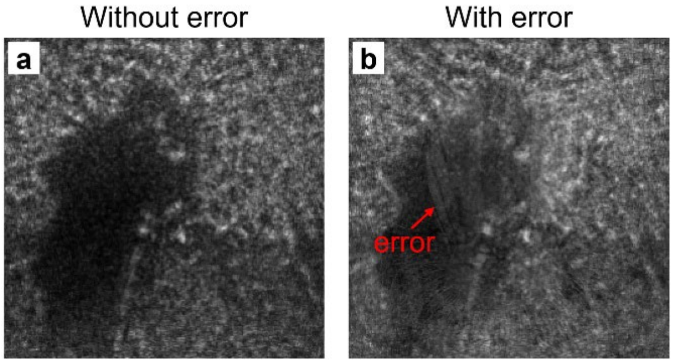

Supplementary Fig. S19 | Comparison of raw images generated by the forward model. (a) The first raw image without error. (b) The first raw image with error.

In step 7, we generate the initial guesses for both the object and the coded surface profile in both the object state and virtual state, as shown in Supplementary Fig. S20.

```
%% Step 7: Generate the initial guess.
112.objectSum = zeros(imSize0,imSize0);
113.for i=1:imNum
114.shiftInitial = exp(-1j*2*pi.*( fx0Grid.*locX(i)/imSize0+ fy0Grid.*locY(i)/imSize0));
115.objectSum = objectSum+ifft2(fft2(sqrt(imRaw(:,:,i))).* shiftInitial);
116.end
117.objectIniGuess = ifft2 (ifftshift (propD1Reverse.* propD2Reverse.*padarray…
(fftshift(fft2(objectSum/imNum)),[imSize0*(mag-1)/2 imSize0*(mag-1)/2])));
118.csIniGuess =ifft2(ifftshift(propD2Reverse.*padarray… (fftshift(fft2(mean(sqrt(imRaw),3))),[imSize0*(mag-1)/2 imSize0*(mag-1)/2])));
119.figure(4);imshow(abs(objectIniGuess),[]);title('Object initial guess')
```

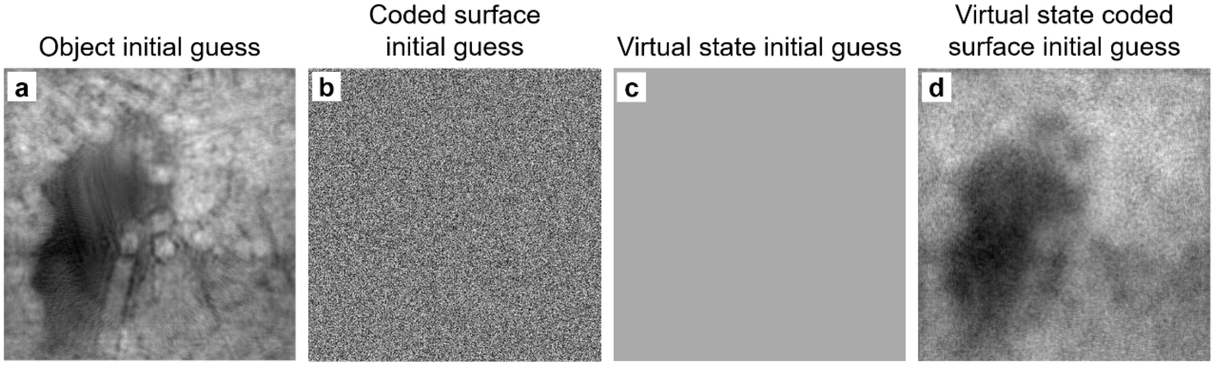

Supplementary Fig. S20 | Initial guesses for the object and coded surface profile in both object and virtual states. (a) Initial guess of the object. (b) Initial guess of the coded surface profile for the object state. (c) Initial guess of the virtual state. (d) Initial guess of the coded surface profile for the virtual state.

In step 8, we recover the object and virtual state using the iterative phase retrieval algorithm. The variable ‘loopNum’ defined in this step determines the number of iterations. The variable alphaO and alphaL are rPIE parameters, typically set to 1 in ePIE. In each iteration, the updated object, virtual state and coded surface are stored

in ‘objectRecovery’, ‘objectRecoveryVirtual’ and ‘csRecovery’, respectively. The final results are shown in Supplementary Fig. S21.

```
%% Step 8: Iterative reconstruction.
120.alphaO = 0.5;
121.alphaL = 0.5;
122.loopNum = 50;
123.objectRecovery = objectIniGuess;
124.csRecovery = cs;
125.objectRecoveryVirtual = ones(size(objectIniGuess));
126.csRecoveryVirtual = csIniGuess;
127.for iLoop = 1:loopNum
128.for i=1:imNum
129.shift = exp(-1j*2*pi.*( fxGrid.*-locX(i)/imSize0+ fyGrid.*-locY(i)/imSize0));
130.objectRecoveryShift = ifft2(fft2(objectRecovery).*shift);
131.objectRecoveryVirtualShift = ifft2(fft2(objectRecoveryVirtual).*shift);
132.objectRecoveryIllum = objectRecoveryShift.*illum;
133.objectRecoveryVirtualIllum = objectRecoveryVirtualShift.*illum;
134.objectWave = ifft2(ifftshift(propD1.*fftshift(fft2(objectRecoveryIllum))));
135.objectWaveVirtual = ifft2(ifftshift(propD1.*fftshift(fft2(objectRecoveryVirtualIllum))));
136.csPlane = objectWave.* csRecovery;
137.csPlaneVirtual = objectWaveVirtual.* csRecoveryVirtual;
138.sensorPlane = ifft2(ifftshift(propD2.*fftshift(fft2(csPlane))));
139.sensorPlaneVirtual = ifft2(ifftshift(propD3.*fftshift(fft2(csPlaneVirtual))));
140.intenSensorPlane = conv2(sum(abs(sensorPlane).^2,3),ones(mag,mag),'same');
141.intenSensorPlaneVirtual = conv2(sum(abs(sensorPlaneVirtual).^2,3),ones(mag,mag),'same');
142.intensity = intenSensorPlane (centerPixel:mag:end,centerPixel:mag:end)+ intenSensorPlaneVirtual
(centerPixel:mag:end,centerPixel:mag:end);
143.ratioMap = sqrt(imRaw(:,:,i))./sqrt(intensity);
144.ratioMap = imresize(gather(ratioMap),mag,'nearest');
145.sensorPlaneUpdate = ratioMap.* sensorPlane;
146.sensorPlaneVirtualUpdate = ratioMap.* sensorPlaneVirtual;
147.csPlaneUpdate = ifft2(ifftshift(propD2Reverse.*fftshift(fft2(sensorPlaneUpdate))));
148.csPlaneVirtualUpdate = ifft2(ifftshift(propD3Reverse.*fftshift(fft2(sensorPlaneVirtualUpdate))));
149.objectWaveUpdate = objectWave + (csPlaneUpdate - csPlane) .*…
(conj(csRecovery) ./ (alphaO.*max(max(abs(csRecovery).^2))+(1-alphaO).*(abs(csRecovery)).^2)) ;
150.csRecovery = csRecovery + sum((csPlaneUpdate - csPlane) .* (conj(objectWaveUpdate) ./ (alphaL.*…
max(max(sum(abs(objectWaveUpdate).^2, 3)))+(1-alphaL).*(sum(abs(objectWaveUpdate).^2, 3)))), 3);
151.objectWaveVirtualUpdate = objectWaveVirtual + (csPlaneVirtualUpdate - csPlaneVirtual) .*…
(conj(csRecoveryVirtual) ./ (alphaO.*max(max(abs(csRecoveryVirtual).^2))+…
(1-alphaO).* (abs(csRecoveryVirtual)).^2)) ;
152.objectRecoveryIllumUpdate = ifft2(ifftshift(propD1Reverse.*fftshift(fft2(objectWaveUpdate))));
153.objectRecoveryVirtualIllumUpdate =…
ifft2(ifftshift(propD1Reverse.*fftshift(fft2(objectWaveVirtualUpdate))));
154.objectRecoveryShift = objectRecoveryShift + sum((objectRecoveryIllumUpdate -… objectRecoveryIllum) .*
(conj(illum) ./ (alphaL.*max(max(sum(abs(illum).^2, 3)))+…
(1-alphaL).*(sum(abs(illum).^2, 3)))), 3);
155.objectRecoveryVirtualShift = objectRecoveryVirtualShift + sum((objectRecoveryVirtualIllumUpdate -…
objectRecoveryVirtualIllum) .* (conj(illum) ./ (alphaL.*max(max(sum(abs(illum).^2, 3)))+…
```

```
(1-alphaL).*(sum(abs(illum).^2, 3)))), 3);
shiftReverse = exp(-1j*2*pi.*( fxGrid.*locX(i)/imSize0+ fyGrid.*locY(i)/imSize0));
 objectRecovery = ifft2(fft2(objectRecoveryShift).* shiftReverse);
 objectRecoveryVirtual = ifft2(fft2(objectRecoveryVirtualShift).* shiftReverse);
end
figure(5);
subplot(231);imshow(abs(objectGT),[]);title({'Object amplitude', '(Ground truth)'})
subplot(232);imshow(abs(objectRecovery),[]);title({'DART recovered amplitude', 'with virtual state'})
subplot(233);imshow(abs(objectRecoveryVirtual),[]);title({'virtual state amplitude'})
subplot(234);imshow(angle(objectGT),[]);title({'Object phase', '(Ground truth)'})
subplot(235);imshow(angle(objectRecovery),[]);title({'DART recovered phase', 'with virtual state'})
subplot(236);imshow(angle(objectRecoveryVirtual),[]);title({'virtual state phase'})
pause(0.1)
end
```

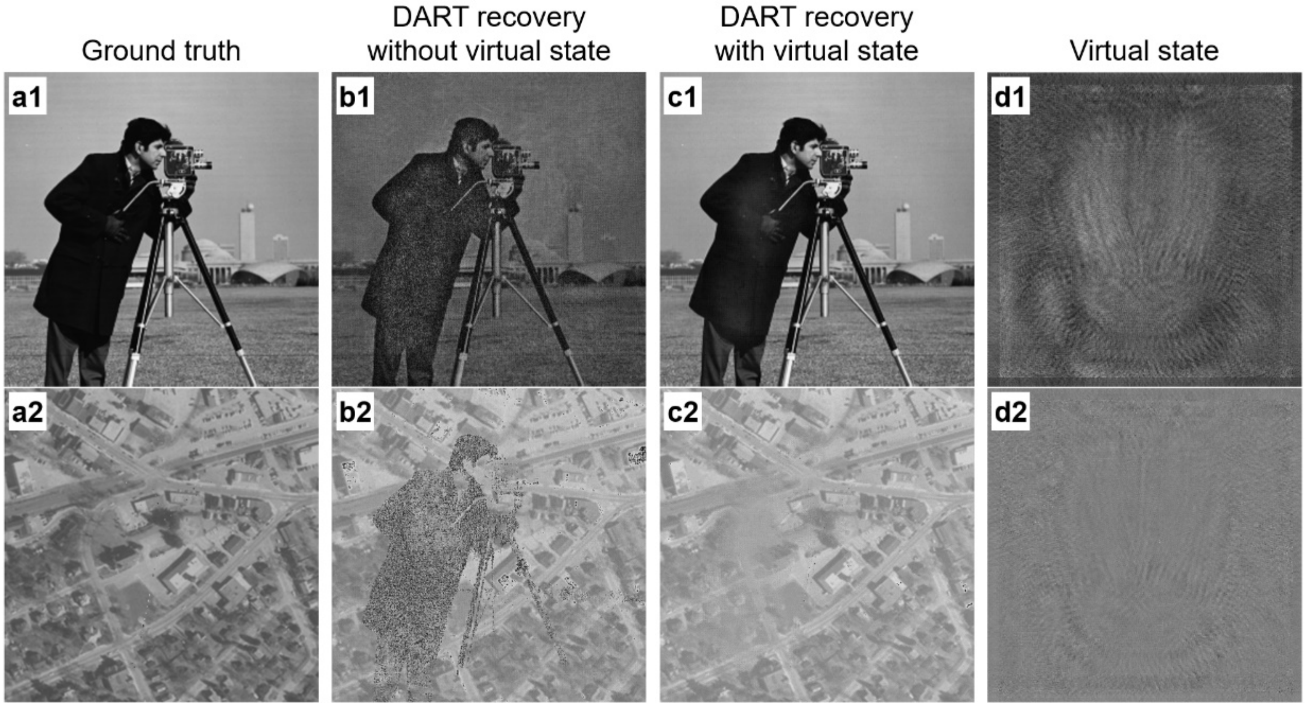

Supplementary Fig. S21 | Comparison of single state reconstruction and object recovery with virtual state correction. (a1, a2) Ground truth amplitude and phase images for reference. (b1, b2) DART recovery without virtual state correction, showing incomplete reconstructions with prominent artifacts in both amplitude and phase. (c1, c2) DART recovery with virtual state correction, exhibiting improved amplitude and phase reconstructions with significantly fewer artifacts. (d1, d2) Virtual state recovery, highlighting corrections for the error.

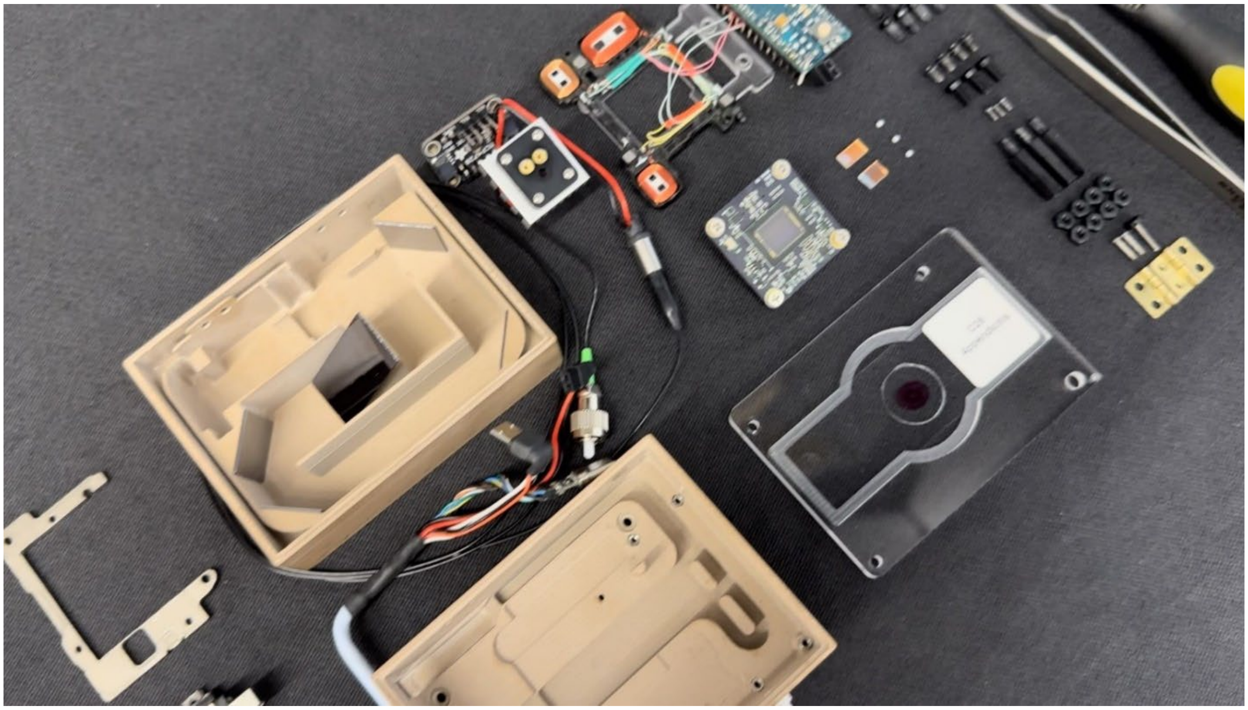

Supplementary Video S1 | Assembly of the DART prototype. This video walks through the assembly process of the DART from the installation of individual components to the final assembly. The video highlights key steps such as mounting the UV-enhanced mirrors, positioning the DUV LEDs, and integrating the motion constraint rails. It also illustrates the addition of the voice coil actuator system, which allows precise control over sensor movement. By the end of the video, the compact and handheld DART system is fully assembled and ready for imaging application.

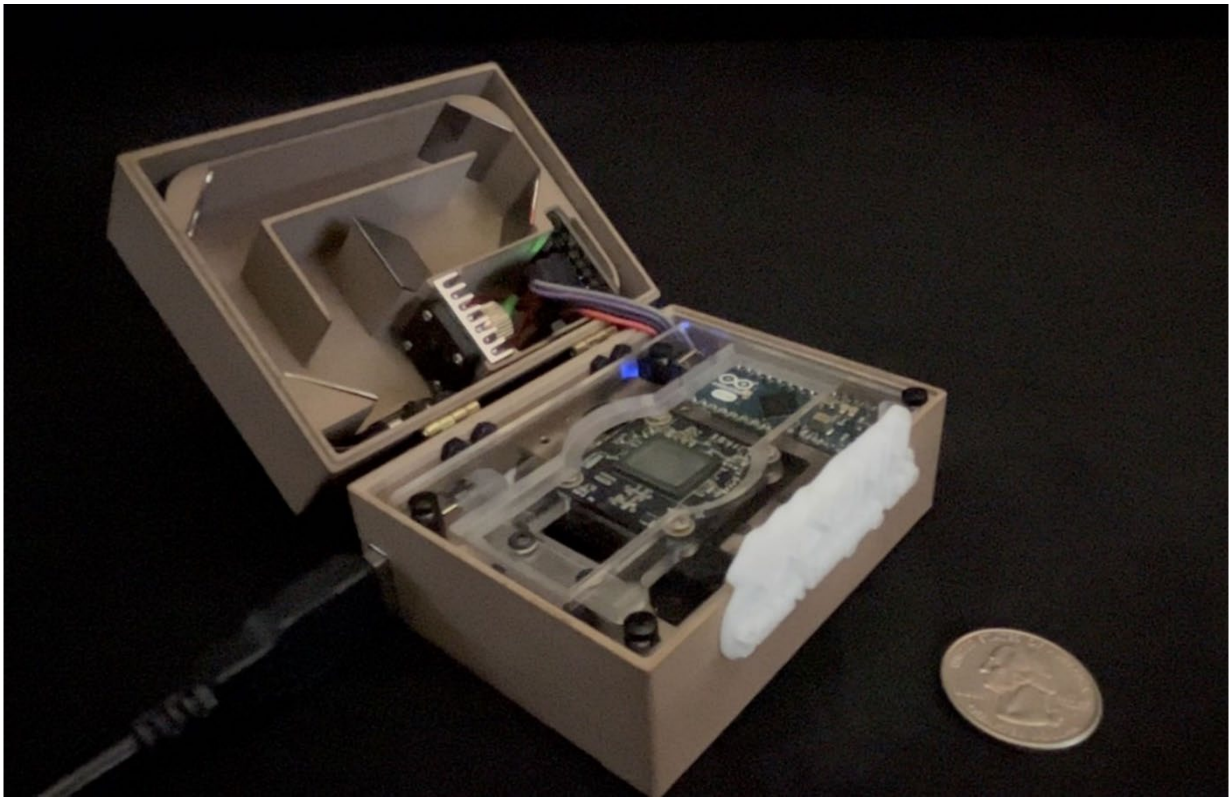

Supplementary Video S2 | Operation of the DART device. This video demonstrates the operational workflow of the DART system, showcasing the user-friendly process of sample placement, activation of light sources, and real-time diffraction image acquisition. The smooth movement of the sensor, enabled by the voice coil actuator system, is shown in action, reflecting the fine mechanical control that ensures high-resolution imaging. This video provides insight into how DART functions in practice.

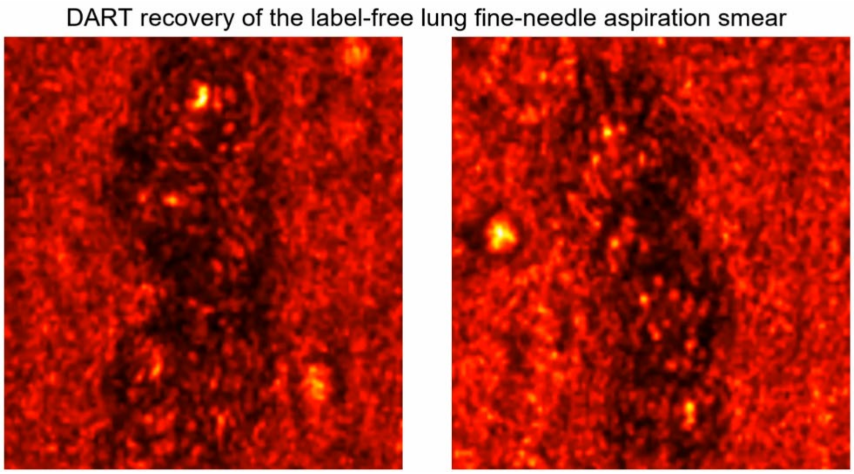

Supplementary Video S3 | DART reconstruction comparison with and without the virtual state. This video compares two DART reconstructions of an unstained lung fine-needle aspiration (FNA) smear: one with virtual state correction and one without. The video clearly demonstrates the improvement in image fidelity when using the virtual state, which isolates and removes system artifacts, particularly those caused by imperfections in temporal coherence or multi-reflection fringes. By comparing the reconstructions, the video underscores the importance of the virtual error-bin state in achieving clearer, more accurate imaging results.
